# Obturator hernia mimicking recurrent femoral hernia: A diagnostic challenge in an elderly patient (case report)

**DOI:** 10.1016/j.ijscr.2025.112079

**Published:** 2025-10-20

**Authors:** Boubker Idrissi Kaitouni, Fernando Nantote, Stephane Ayee, Zaynab Bellamlik, Mbarek Yaka

**Affiliations:** a Digestive Surgical Department C, Centre Hospitalier Ibn Sina, Rabat, Morocco; b Digestive Surgical Department, Mohammed V Military Hospital, Rabat, Morocco; c Faculty of Medicine and Pharmacy, Mohammed V University in Rabat, Morocco

**Keywords:** Obturator hernia, Obstruction, Laparotomy, Case report

## Abstract

**Introduction:**

Obturator hernia is a rare cause of bowel obstruction, predominantly affecting elderly women. Its non-specific symptoms often delay diagnosis, increasing the risk of complications.

**Case presentation:**

We report the case of an 86-year-old woman presenting with signs of intestinal obstruction. CT imaging identified a strangulated right obturator hernia without ischemia, managed successfully via laparotomy. This case report has been reported in line with the SCARE 2025 checklist (Kerwan et al., 2025 [1]).

**Discussion:**

While laparoscopy is increasingly used in selected cases, open surgery remains appropriate in emergencies or when technical conditions are unfavorable. Our decision for laparotomy was guided by marked bowel distension and respiratory constraints.

**Conclusion:**

This case highlights the diagnostic challenge of obturator hernia, particularly in elderly patients with prior hernia history, where atypical presentations may mimic other conditions. Tailored surgical strategies, guided by clinical and intraoperative findings, are essential to optimize outcomes in such high-risk cases.

## Highlights

• Strangulated obturator hernia is an uncommon but critical cause of small bowel obstruction, typically seen in elderly, thin women.
• Its diagnosis is often delayed due to vague symptoms and non-specific imaging findings. Early surgical management is key to reducing morbidity and improving prognosis.
• This case reports an 85-year-old woman presenting with small bowel obstruction caused by a strangulated obturator hernia. Surgical management via midline laparotomy led to a favorable intraoperative outcome

## 1 Introduction

Obturator hernia is an uncommon clinical entity, constituting less than 1 % of all abdominal wall hernias [2]. It predominantly affects elderly, emaciated, and multiparous women, and is frequently characterized by vague, non-specific symptoms, which contribute to delayed or missed preoperative diagnosis [2,3]. Often identified only when it progresses to mechanical bowel obstruction, this condition is associated with a high risk of morbidity and mortality if not promptly managed [3,4]. CT imaging remains the cornerstone of accurate and timely diagnosis [3]. We herein present a case of strangulated obturator hernia in an 86-year-old female patient, highlighting the diagnostic challenges of atypical presentations, particularly in patients with a history of prior hernia repairs, and the importance of tailored surgical management. This case report has been reported in line with the SCARE checklist [1].

## 2 Case presentation

We report the case of an 86-year-old woman with a past medical history of well-controlled hypertension on dual therapy. Five months prior to her current admission, she presented to the emergency department with signs of bowel obstruction secondary to a strangulated left femoral hernia. She underwent emergency surgery via elective laparotomy, during which hernia repair was performed without bowel resection, as the small intestine was found to be viable.

She was readmitted five months later with symptoms consistent with a new episode of intestinal obstruction. She reported a four-day history of absence of bowel movements and flatus, persistent vomiting, and pain localized to the root of the right thigh. On examination, she was hemodynamically stable but tachypneic at 28 breaths per minute. Her body mass index was 22, and her performance status was classified as WHO grade 2 (indicating slight limitation in physical activity but ambulatory and capable of self-care) and ASA class II (indicating mild systemic disease without substantive functional limitations). Abdominal examination revealed marked distension, mild diffuse tenderness, tympanic sounds on percussion, and an empty rectal ampulla. No external hernia orifices were palpable.

**Fig. 1:**
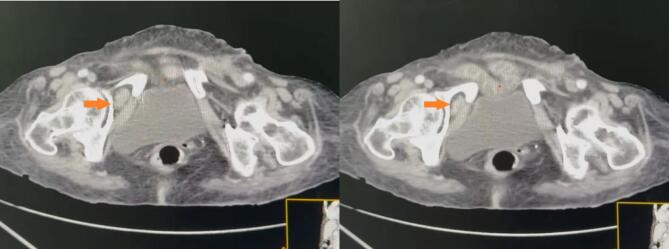
A, B: Axial CT scan showing a right-sided obturator hernia.

**Fig. 2:**
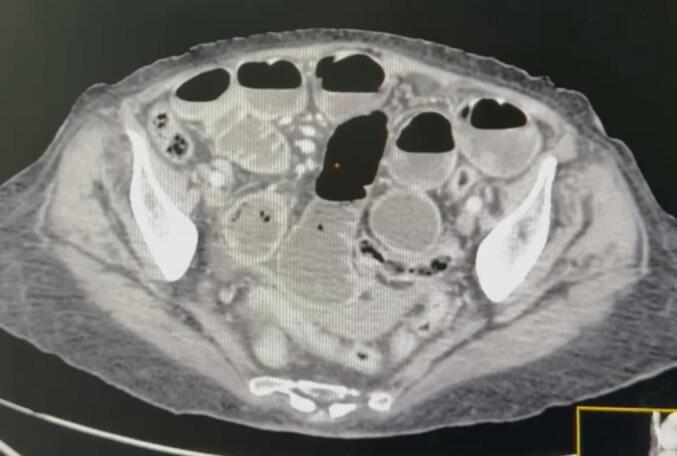
Axial CT scan demonstrating significant small bowel distension.

A contrast-enhanced abdominopelvic CT scan and complete blood workup were performed. Senior radiological review confirmed the diagnosis of small bowel obstruction caused by a strangulated right obturator hernia, with no radiologic signs of bowel ischemia (Fig. 1, Fig. 2). Laboratory tests revealed a systemic inflammatory response, with a C-reactive protein level of 170 mg/L, neutrophil count of 16,700/mm^3^, and hypokalemia at 2.3 mmol/L. Surgery was indicated, and the intensive care team was contacted for perioperative management.

**Fig. 3:**
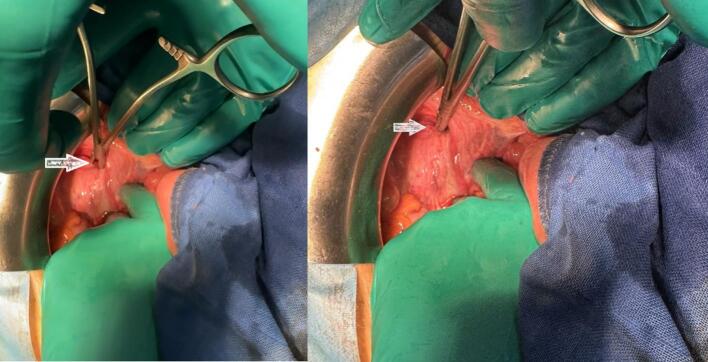
A, B: Intraoperative view of the obturator foramen as the site of bowel entrapment.

**Fig. 4:**
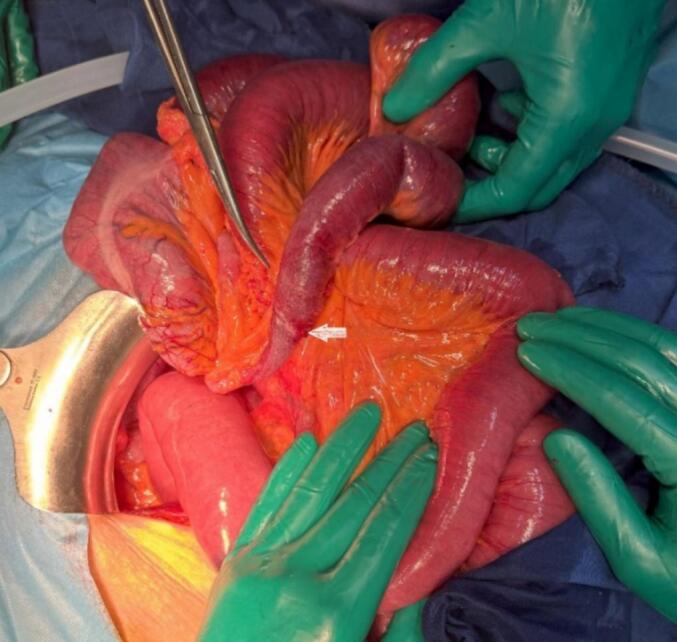
Intraoperative image revealing a transition point at the terminal ileum.

After discussion with the anesthesia team, a conventional open approach was chosen over laparoscopy due to marked bowel distension, restrictive respiratory pattern, advanced age, and a history of prior abdominal surgery. A lower midline laparotomy was performed, revealing significant small bowel distension and entrapment of the terminal ileum within the right obturator canal (Fig. 3, Fig. 4). The bowel was viable, and a moderate amount of clear peritoneal fluid was noted, with no signs of ischemia. Surgical intervention involved retrograde decompression of the bowel, followed by primary repair of the obturator defect using 2-0 Vicryl sutures (a resorbable suture chosen due to the absence of prosthetic material availability and the need for rapid closure in an emergency setting).

The postoperative course was uneventful, with return of bowel function by postoperative day two. Oral intake was reintroduced progressively starting on the first postoperative day. The patient was discharged on postoperative day 4 after correction of hypokalemia. At the 15-day follow-up, she was in good condition and expressed satisfaction with the care provided.

## 3 Discussion

Although rare, obturator hernia remains a potentially serious cause of acute intestinal obstruction in elderly patients. It is often-silent progression until strangulation contributes to the challenge of early diagnosis. Recent series emphasize the importance of maintaining a high index of suspicion in cases of bowel obstruction without clear etiology—particularly in elderly women, even in the absence of suggestive clinical signs [2,3]. In this case, the patient's history of a prior femoral hernia repair initially led to a suspicion of recurrence, underscoring the diagnostic pitfall of atypical presentations mimicking other hernia types.

In this setting, imaging plays a pivotal role not only in diagnosis but also in guiding therapeutic decision-making [3]. Contrast-enhanced abdominopelvic CT is currently regarded as the gold standard for diagnosing obturator hernias. It enables direct visualization of the herniated bowel loop through the obturator canal, while also assessing intestinal viability and identifying potential ischemic complications [4,5]. In our case, a second reading of the CT scan by a senior radiologist proved decisive in confirming the diagnosis.

The treatment of obturator hernia is exclusively surgical. In emergency situations, management involves reduction of the strangulated bowel loop and repair of the hernia defect. The choice between open surgery and laparoscopy depends on the clinical context, available resources, and institutional protocols. In cases of strangulation, the open approach is often favored, as it allows for complete exploration of the peritoneal cavity and facilitates intestinal resection when necrosis is present [5,6]. In our case, the use of 2-0 Vicryl sutures, which are resorbable, was driven by the unavailability of prosthetic material and the need for rapid closure in an emergency setting. While non-absorbable sutures or prosthetic mesh are typically preferred to minimize recurrence risk, as reported in studies such as Miladinovic et al. [7], the absence of ischemia and the patient's stable postoperative recovery supported the adequacy of this approach in this context.

The laparoscopic approach has been increasingly reported in recent years, particularly in selected clinical contexts [6]. It offers several advantages, including improved postoperative comfort, shorter hospital stays, and faster recovery [6,7]. Studies, such as Liu et al. [6] and Doden et al. [8], highlight the feasibility of laparoscopic repair in stable patients with favorable operative conditions. However, in our case, significant bowel distension and respiratory constraints necessitated an open approach. Furthermore, systematic inspection of the obturator foramen during laparoscopic hernia repairs is critical, as it can prevent missed diagnoses in patients with atypical presentations [6].

## 4 Conclusion

Obturator hernia, though uncommon, represents a diagnostic and therapeutic challenge due to its non-specific presentation and potential to mimic other conditions. This case underscores the importance of maintaining a high index of suspicion in elderly patients with bowel obstruction, particularly those with prior hernia repairs. Advances in imaging, particularly contrast-enhanced CT, have improved diagnostic accuracy, while surgical management must be tailored to the patient's clinical condition and intraoperative findings. The use of resorbable sutures in this case highlights the need for context-specific decision-making, balancing immediate surgical constraints with long-term outcomes.

## Author contribution

BIK designed the paper.

SA and FN collected the data.

BIK and ZB wrote the first draft of the manuscript.

BIK participated in the article design and critically reviewed the manuscript.

MY and FN critically reviewed the manuscript.

All authors approved the final version of the manuscript.

## Consent

Written informed consent was obtained from the patients for publication of this case report and accompanying images.

## Ethical approval

Ethical approval for this study was provided by the Ethical Committee.

## Guarantor

Boubker Idrissi Kaitouni.

## Research registration number

Not applicable.

## Funding

N/A.

## Conflict of interest statement

N/A.
